# Crosstalk between metabolism and cell death in tumorigenesis

**DOI:** 10.1186/s12943-024-01977-1

**Published:** 2024-04-04

**Authors:** Shichao Yang, Caden Hu, Xiaomei Chen, Yi Tang, Juanjuan Li, Hanqing Yang, Yi Yang, Binwu Ying, Xue Xiao, Shang‑Ze Li, Li Gu, Yahui Zhu

**Affiliations:** 1 School of Medicine, Chongqing University, Chongqing, 400030 P. R. China; 2 Molecular Medicine Diagnostic and Testing Center, Chongqing Medical University, Chongqing, P. R. China; 3 Department of Pathology, College of Basic Medicine, Chongqing Medical University, Chongqing, P. R. China; 4 Department of breast and thyroid surgery, Renmin hospital of Wuhan University, Wuhan, 430060 P. R. China; 5 Institute of Pathology and Southwest Cancer Center, The First Affiliated Hospital, Key Laboratory of Tumor Immunopathology, Third Military Medical University (Army Medical University, Ministry of Education of China, Chongqing, 400038 P. R. China; 6 Department of Laboratory Medicine/Clinical Laboratory Medicine Research Center, West China Hospital, Sichuan University, Chengdu, Sichuan Province 610041 P. R. China; 7 Department of Gynecology and Obstetrics, West China Second University Hospital, Sichuan University, Chengdu, P. R. China; 8 Key Laboratory of Birth Defects and Related Diseases of Women and Children (Sichuan University), Ministry of Education, West China Second Hospital, Sichuan University, Chengdu, P. R. China

**Keywords:** Tumor metabolism, Cell death, Apoptosis, Pyroptosis, Ferroptosis, Cuproptosis, Autophage, Tumor microenvironment

## Abstract

It is generally recognized that tumor cells proliferate more rapidly than normal cells. Due to such an abnormally rapid proliferation rate, cancer cells constantly encounter the limits of insufficient oxygen and nutrient supplies. To satisfy their growth needs and resist adverse environmental events, tumor cells modify the metabolic pathways to produce both extra energies and substances required for rapid growth. Realizing the metabolic characters special for tumor cells will be helpful for eliminating them during therapy. Cell death is a hot topic of long-term study and targeting cell death is one of the most effective ways to repress tumor growth. Many studies have successfully demonstrated that metabolism is inextricably linked to cell death of cancer cells. Here we summarize the recently identified metabolic characters that specifically impact on different types of cell deaths and discuss their roles in tumorigenesis.

**Supplementary Information:**

The online version contains supplementary material available at 10.1186/s12943-024-01977-1.

## Introduction

Metabolism usually refers to a series of biochemical reactions, which is divided into two categories: catabolism and anabolism [1]. Different metabolic reactions coordinate with each other in vivo to jointly maintain the vital functions of the normal organism. Various substances produced can play a role in signal transductions and participate into various biological processes of the organism. For example, cAMP acts as an important second messenger [2, 3]. According to the differences in types of substrates generated or consumed, metabolism can be divided into carbohydrate metabolism, lipid metabolism, amino acid metabolism, nucleotide metabolism, etc. These different metabolic processes are not only involved in the maintenance of body homeostasis, but also associated with the development of diseases. For example, disorders of purine metabolism increase uric acid levels, and the resulted excess uric acidcauses inflammation and further leads to joint swelling [4, 5]. Overall, metabolicdisorders can induce many diseases such as diabetes, hyperlipoproteinemia, hypercalcemia, etc. The metabolism of cancer cells is different from that of normal cells [6]. For example, the Warburg effect is a metabolic process that exists in tumors. It is recognized as a way to gain energy through glycolysis even in the presence of abundant oxygen [7]. Metabolism dysregulation is one of the main hallmarks for the proliferation and invasion of tumors. In the absence of energy, cells may experience a series of function disordersthat even lead to cell death [8].

Cells have a life span and are subject to aging and death, and cell death is inevitable. Based on the different trigger mechanisms and processes, cell death can be divided into apoptosis, necrosis, necroptosis, ferroptosis, pyroptosis, cuproptosis, autophagy, etc. These forms of cell deaths affect the specifically relative cells on their physiological activities and survivals under many different mechanisms. For example, autophagy removes the damaged or senescent organelles by forming autophagosomes [9], while apoptosis is dependent on apoptosomes formation [10]. Cell death can also occur under some stress conditions that are not conducive to cell growth, such as hypoxia, lack of nutrients, or external stimuli. The survival of tumor cells can be affected by targeting cell death-related genes and can further be developed into a strategy for tumor therapy. For example, autophagy can be promoted by suppressing the autophagy-related mTOR pathway [11], thus supporting cancer cells growth. Autophagy not only supports tumor growth, but also can play the role as a tumor suppressor [12]. Under the suppression of MCOLN1/TRPML1, autophagy inhibits tumor metastasis through the TP53/p53 pathway [13]. Targeted cell death is currently used alone or in combination with other types of tumor therapies for cancer treatments [14, 15]. In recent years, as part of the cell death derives from metabolic stress and another part can be involved in metabolic regulation, the relationship between cell death and metabolism has received increasing attention in tumor development and treatment.

The tumor microenvironment is also essential for the development of tumors. The tumor microenvironment is crucial for tumor growth, so changes occurring in this microenvironment largely affects tumor cell survival and growth [16]. The microenvironment is rich and diverse and contains various tumor growth factors, extracellular matrices, and many other types of cells such as immune cells and fibroblasts [17]. These substances participate in tumor growth, development and immune processes [18]. Because of this, the tumor microenvironment has become one of the important targets for tumor therapy. Tumor immunotherapy is a very effective method that includes immune checkpoint therapy and CAR-T therapy, etc. Tumor cells can evade immunological cytotoxicity and immunological surveillance through immune checkpoints such as PD-1/PD-L1 or CTLA-4 [19–21]. For example, PD-1/PD-L1 receptor-ligand interactions are activated to suppress the immune function of T cells in tumors [22–24]. This process is called tumor immune escape [25, 26]. The tumor microenvironment, as the location where tumor cells exist, is involved in various tumor regulatory processes [27]. Immune checkpoint molecules and multiple immune cells, as well as other types of immune molecules present in the tumor microenvironment, make the tumor microenvironment important for immune checkpoint therapy or other immunotherapies [28, 29].

In this review, we will discuss how various metabolic pathways affect different cell death models, mainly focus on the impacts on tumor growths through some crosstalk, which will shed light on the possible connections between metabolic pathways and cell death models within tumor microenvironment. We also hope it will further provide some insights that may help readers investigate the relationships between metabolism and cell death in tumors.

### Cell apoptosis affacted by metabolic activities and substrates during tumorigenesis

Apoptosis is one type of programmed cell death that is morphologically characterized by cell shrinkage, compact intracellular arrangement of organelles, nuclear division, and the appearance of apoptotic body in the cytoplasm [30]. Apoptosis is usually classified into two types: the intrinsic pathway and the extrinsic pathway. The intrinsic pathway of apoptosis, also known as the mitochondrial apoptotic pathway, is caused by endogenous apoptotic signals such as endoplasmic reticulum stress, DNA damage, etc. In this pathway, modification of mitochondria structure and function causes apoptosis [30]. The extrinsic pathway of apoptosis is mediated by cell membrane death receptors and exogenous ligands [31]. Apoptosis requires the mediation of a series of key molecules, among which Bcl-2 family proteins and caspase family proteins, etc. play crucial roles. The Bcl-2 family members have different roles in apoptosis. Protein members such as BAX, and BAK have a pro-apoptotic effect, while BCL-2 and BCL-X_L_ have an inhibitory effect [32]. Caspase proteins such as caspase-9 gain their activity through the cleavage of apoptosome, then involve into the hydrolysis of various intracellular proteins.

**Fig. 1:**
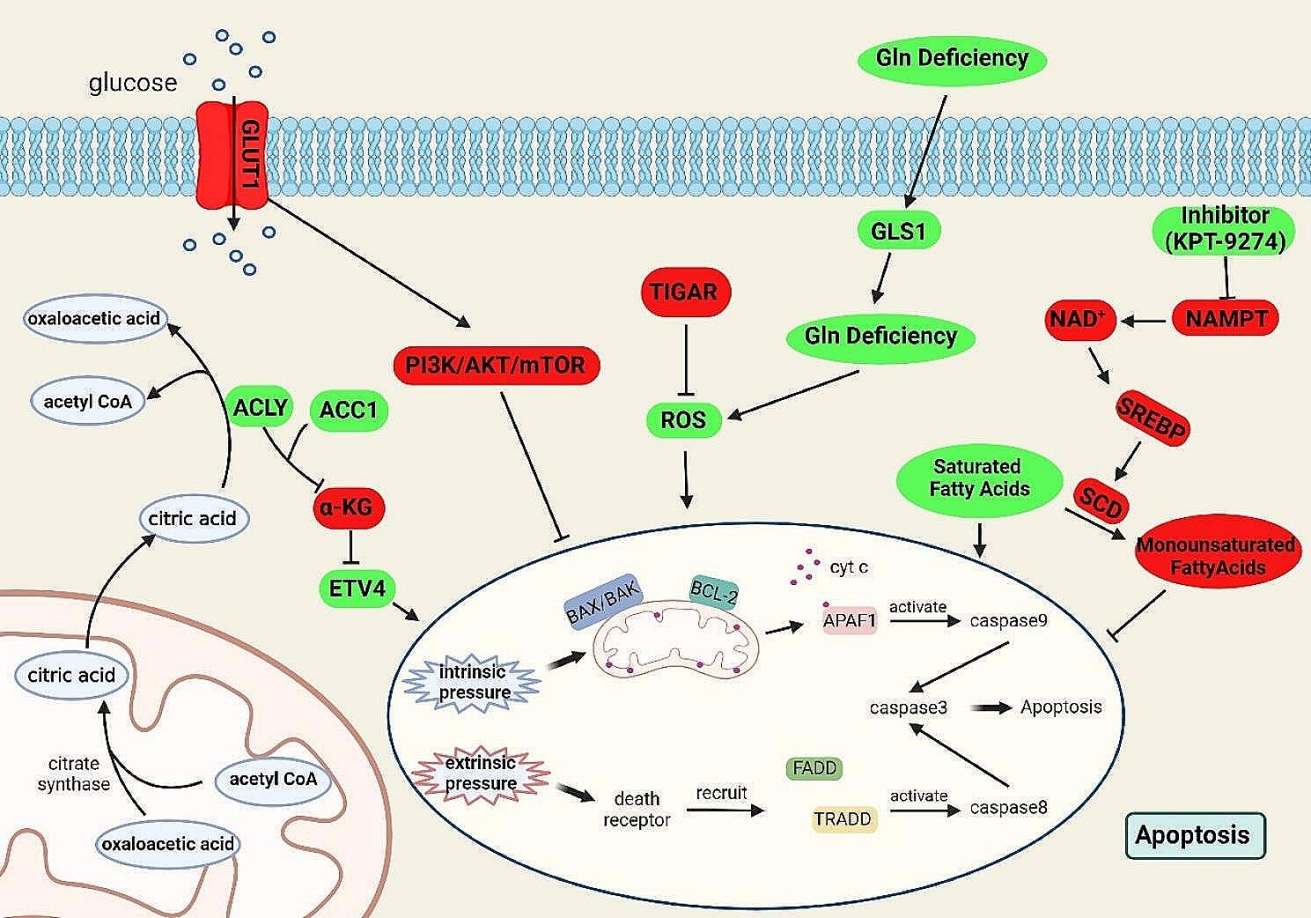
Metabolites, metabolic pathways and related metabolic genes that play the roles in apoptosis. Deficiencies of various substances involved in metabolism affect the relevant metabolic pathways and apoptosis. Glycolysis can be inhibited in the presence of Glut1 deficiency, which promotes the development of apoptosis. ACLY, a key enzyme involved in the conversion of citric acid to oxaloacetate and acetyl CoA, works with ACC1, an important enzyme in the process of acetyl-CoA production, to regulate the content of α-KG and promote ETV4, which in turn promotes apoptosis. ROS usually promotes apoptosis. When TIGAR inhibits the important oxidative ROS, apoptosis can be suppressed. Gln deletion synergizes with GLS1 to promote ROS-related apoptosis. Inhibition of NAMPT prevents the conversion of saturated fatty acids to monounsaturated fatty acids and promotes apoptosis. The red boxes represent negative regulators and the green boxes represent positive regulators

One of the most significant changes for tumor metabolism is aerobic glycolysis. Therefore, regulating the enzymes and proteins in glucose metabolism to change energy production rate can effectively modulate tumor growth. Regulation of the activity and function of glucose transporter (GLUT) proteins indeed influences apoptosis and further affects tumor growth [33]. GLUT1 regulates the PI3K/AKT signaling pathways to adjust tumor proliferation and apoptosis [34, 35]. AKT and p53 mutations exist in many different tumor types. Interactions between AKT and p53 in tumor cells affects apoptosis [36]. The p53-inducible gene TIGAR regulates the intracellular fructose diphosphate level. It also reduces the contents of reactive oxygen species to protect cells from ROS-related apoptosis [37]. In similar with the effects of deprivation and glucose metabolism blockage to cause insufficient energy production, abnormal activities of glucose metabolism-related enzymes can also cause this problem to reach to the same levels. For instance, pyruvate kinase, a key enzyme in glycolysis, inhibits apoptosis by supporting glycolysis. Besides repressing key enzymes during glycolysis, key enzymes in the citrate pyruvate cycle such as ATP citrate lyase (ACLY) also regulate apoptosis [38, 39]. In addition, ACLY can be deubiquitinated in the presence of USP30, which regulates the IKKβ-USP30-ACLY signaling axis and further effectively modulates lipid synthesis [40]. These studies show that correlations exist between multiple different metabolic processes and apoptosis. During the same time, many types of carbohydrate metabolism processes may get interactions through certain common enzymes or metabolites (Fig. 1) [41, 42]. Although it has not been fully proved that apoptosis can happen in all types of known carbohydrate metabolisms, it is still expected that the additional metabolic entry points for both regulating apoptosis in tumor cells and further influencing the ongoing tumorigenesis will be finally identified-after the specificities of interactions for different carbohydrate metabolism processes will be clarified.

In addition to carbohydrates, amino acids are also important nutrients as well as important regulatory factors in the organism and tumor development. Similar to glucose, amino acid intakes can also influence apoptosis [43, 44]. Glutamine helps tumors to resist apoptosis, while its deficiency can induce apoptosis [45–47]. Glutamine deficiency and GLS filamentous polymers in cells together lead to asparagine deficiency and ROS-related apoptosis [47]. Other types of amino acids such as proline are also involved in the regulation of tumor cell apoptosis [48, 49]. Similar to glucose metabolism, amino acid transporters also adjust apoptosis in tumors, suggesting that uptake of energy or substances can effectively affect apoptosis. L-type amino acid transporter 1 (LAT1, SLC7A5) is upregulated in many tumors and inhibition of LAT1 function makes cancer cells more sensitive to apoptosis [50, 51]. Besides, some essential amino acids such as phenylalanine and methionine are involved in the regulation of apoptosis (Fig. 1) [52].

Palmitic acid is a highly abundant free fatty acid in the human body and is involved in the regulation of apoptosis [53, 54]. Lipids other than palmitic acid are also involved in the regulation of apoptosis. The oxidized low-density lipoprotein (OX-LDL) is closely related to endothelial cell damage and apoptosis [55, 56]. Besides apoptosis, lipids are also associated with tumorigenesis. ACAT1 acetylates GNAPT to regulate lipid metabolism and promotes hepatocarcinogenesis [57]. SREBP, a key factor in lipid synthesis, has been shown to be involved in apoptosis. SREBP and FASN targeting drugs can inhibit lipid synthesis to induce apoptosis in cancer cells [58]. The SREBP-regulated gene SCD is known to involve into apoptosis. Nicotinamide phosphoribosyltransferase (NAMPT) inhibition can influence on the conversion from the saturated fatty acids to the monounsaturated fatty acids as well as on the expression of SCD, which further have an effect on apoptosis [59]. Other members of the lipid family and lipid metabolic processes have also been shown to be involved in the regulation of apoptosis in various contexts [60–62], demonstrating their indispensable role in regulating tumorigenesis.

Usually, apoptosis can be induced based on the relative mechanisms of either promoting or inhibiting the acquisition of energy. Mitochondria is a vital place for oxidative phosphorylation, which is required by both the aerobic oxidation of glucose and the β-oxidation of triglycerides [63]. Besides, mitochondria is also an important site for regulation of endogenous apoptotic pathway. Thus, modulation of structure and function of mitochondria of tumor cells can induce their apoptosis [64, 65]. Taken together, these studies suggest that it is feasible to influence tumor cell apoptosis through metabolism, either by directly reducing nutrient intake or affecting cellular nutrient utilization (Fig. 1). Tumor cell apoptosis regulated by metabolism does not just inhibit tumorigenesis, sometimes it appears to promote tumor growth. We may use this as a starting point to find more methods that can effectively inhibit tumorigenesis.

### The crosstalk between metabolism and necrosis during tumorigenesis

Cell necrosis is defined as a pathological injury that is caused by factors such as physical damage, chemical stimulation or hypoxia. One of the most significant morphological features of necrosis is the rupture of cell membrane [66]. Due to the broken membrane, intracellular inflammatory substances are released into the surrounding environment and further induce an inflammatory response. Necrosis is a very common phenomenon in tumors [67]. Since the formation of blood vessels cannot keep up with a rapid expansion of the tumor tissue volume, a remarkable feature of solid tumors is that the internal tumor tissue is often devoid of oxygen and nutrients, thus making it more prone to necrosis.

**Fig. 2:**
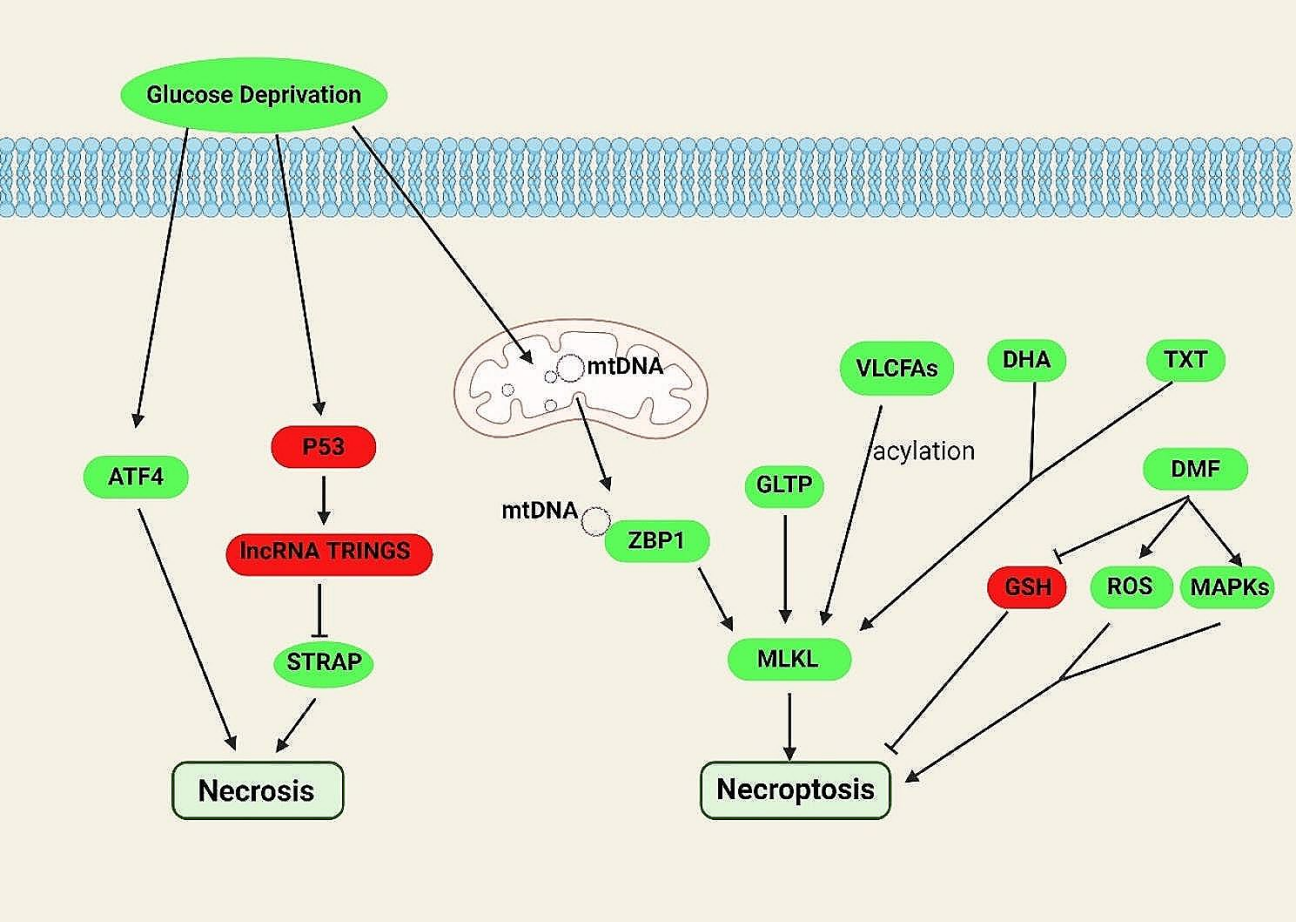
Metabolites, metabolic pathways and related metabolic genes that take part in necrosis and necroptosis. Glucose starvation promotes necrosis through the transcription factor ATF4. In addition, it can act on p53, which regulates necroptosis by affecting the interaction between TRINGS and STRAP. Glucose deprivation also facilitates necroptosis by promoting the binding of mitochondrial DNA and ZBP1 to regulate MLKL, a key substance in the development of necroptosis. DHA supplementation with docetaxel (TXT) promotes necroptosis. As one of the key components of the necrosome that promotes the onset of necroptosis, MLKL function can be facilitated by GLTP. Very long chain saturated fatty acids participate in necroptosis by targeting MLKL. DMF promotes necroptosis by promoting the depletion of GSH, ROS generation and MAPK activation. The red boxes represent negative regulators and the green boxes represent positive regulators

Glucose metabolism is an important regulatory activity for tumors. It affects tumor growth by regulating apoptosis, autophagy, and other different kinds of cell deaths. Inhibition of glucose uptake is an important trigger for tumor necrosis, as cancer cells are more inclined to use glucose for glycolysis to gain energy [68]. During the process, the genes relative to energy metabolism is used as the inducing targets to regulate cell death. The transcription factor ATF4 also plays a role in necrosis that is regulated by glucose deprivation [69]. ATF4 is associated with p53 in different signaling pathways and influences the onset of other types of cell death in tumors [70, 71]. P53 also upregulates the expression of the lncRNA TRINGS in the context of glucose deficiency, allowing the increased TRINGS to bind STRAP for inhibiting STRAP mediated necrotic signaling [72]. Besides low glucose, high glucose levels can also regulate necrosis in many situations (Fig. 2) [73–77], demonstrating the broad role of glucose in regulating necrosis.

Specific types of amino acids can promote tumor necrosis and achieve anti-tumor effects. Such amino acid induced necrosis occurs in prostate tumors [78]. Besides nutrients, other factors also regulate necrosis, such as hypoxia and reactive oxygen species [79, 80]. Hypoxia is an important necrosis inducing factor and converts the glucose deprivation-induced necrosis into AKT-dependent apoptosis [81]. As energy deficiency itself is one critical reason for tumor necrosis, we can curb the uptake and utilize of energy substances to induce necrosis [82, 83]. As a form of cell death that can be modulated by energy stress, it is feasible to influence necrosis in tumor cells through modulating energy-generating or depleting pathways.

### The function of metabolism and necroptosis during tumorigenesis

Necrosis was once not considered to be regulated by genetics, but in subsequent studies, necrosis has been discovered as a gene-regulated cell death and designated necroptosis. Necroptosis is triggered by many protein kinases, including RIPK3, MLKL as well as other critical kinases [84]. Apoptosis-inducing receptors such as FAS, TNF receptor 1 (TNFR1), TNFR2, etc. also play a role in necroptosis. In addition, immune molecules associated with damage-associated molecular patterns (DAMP) stimulates the recognition receptors (PRRs) and leads to necroptosis [85]. Necroptosis as the regulated cell death mode is studied for its application in tumor therapy [15]. Necroptosis associated ZBP1 is regulated by the RNA editing enzyme ADAR1, thus affecting the actual efficacy of immune checkpoint blockade therapy [86]. During tumor prognosis, it has been shown that necroptosis promotes tumor repopulation after the treatment through RIP1/RIP3/MLKL/JNK/IL8 signaling pathway (Fig. 2) [87].

MLKL is one of the key regulators of necroptosis. Several studies have already uncovered the link between MLKL and energy stress, which provides good concept for the metabolic regulation of necroptosis. For example, overexpression of GLTP, a protein involved in the transport of sphingomyelin, induces phosphorylation of MLKL and leads to necroptosis [88]. Meanwhile, after glucose deprivation, ZBP1 is found to activate MLKL and promotes necroptosis in breast cancer cells [89]. Some substances, such as the members of the lipid family, can individually affect necroptosis. Docosahexaenoic acid (DHA), a member of the lipid family, is also related to necroptosis. DHA supplementation with docetaxel (TXT) promotes necroptosis in breast cancer cells [90]. Very long chain saturated fatty acids can also participate in the induction of necroptosis by regulating protein acylation [91]. In addition to the lipid, necroptosis with glutathione participation has also been reported. Dimethyl fumarate (DMF) can induce necroptosis by depleting GSH in colon cancer cells [92]. Moreover, AMPK of glucose dependent kinase regulates necroptosis and tumorigenesis through the activations of RIPK3 [93]. In addition, RIPK3 also links energy metabolism to necrosis and apoptosis [94], suggesting that energetic factors play an important role in RIP3-associated necroptosis. RIPK3 has been studied broadly as a crucial regulator of programmed cell death during tumorigenesis [95–97], and considered as an effective target for blocking tumorigenesis.

### The metabolism and ferroptosis in cancer

Ferroptosis is an iron-dependent programmed cell death, which is mainly manifested by extensive peroxidation of lipid bilayers. Unlike other cell death processes, ferroptosis is dependent on iron accumulation and lipid peroxidation [98]. Ferrous irons play a critical role in promoting phospholipid peroxidation, which in turn enhances ferroptosis. Phospholipids are also over-oxidized under the actions of reactive oxygen species, which cause irreversible damage to the cell membrane [99].

**Fig. 3:**
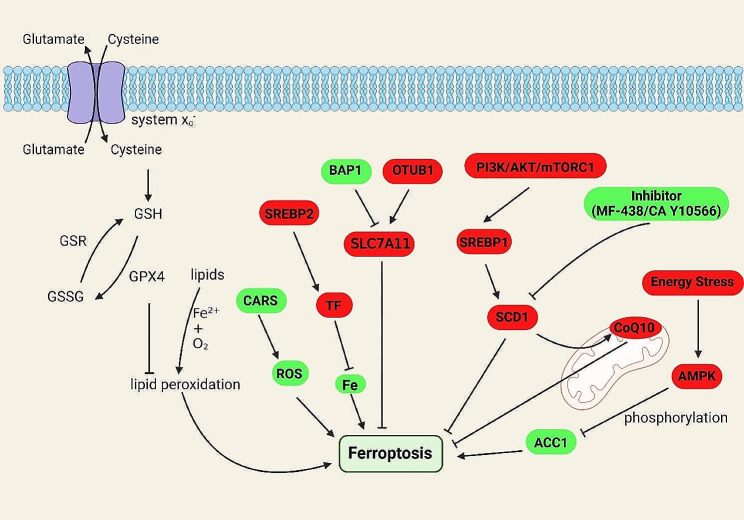
Metabolites, metabolic pathways and related metabolic genes that play the roles in ferroptosis. Lipid oxidation is an important process in ferroptosis and requires the participation of iron. Upregulation of ROS levels by CARS promote ferroptosis, while downregulation of iron ion levels by SREBP2 and TF inhibit ferroptosis. Besides, PI3K/AKT/mTORC signaling pathway activates lipid synthesis-related SREBP1 and SCD1, then affect lipid synthesis to inhibit ferroptosis. While inhibition of SCD1 can influence CoQ10 which locate on the mitochondrial electron transport chain and promote ferroptosis. BAP1 promotes ferroptosis by inhibiting the cystine transport-related SLC7A11, while OTWB1 exerts the opposite effect. Energy stress regulates ACC1 via AMPK, reducing ACC1 activity and inhibiting ferroptosis. The red boxes represent negative regulators and the green boxes represent positive regulators

Both substances and key enzymes of lipid metabolism have been found to participate in the occurrence and development of tumors through affecting ferroptosis [100, 101]. Lipid family members such as polyunsaturated fatty acids are known to induce ferroptosis [102], suggesting that it may be feasible to influence both ferroptosis and ferroptosis-related tumor growth through regulating lipid content. In addition, enzymes of fatty acid metabolism and lipid transport also play a role in the regulation of tumor ferroptosis. It is well demonstrated that ferroptosis and further tumorigenesis can be regulated by lipid metabolism alteration. Stearoyl-CoA desaturase 1 (SCD1), a key enzyme in the conversion of saturated fatty acids to monounsaturated fatty acids, plays an important role in regulating the lipid metabolism related to ferroptosis [103]. Inhibition of stearoyl-CoA desaturase reduces the levels of membrane antioxidant CoQ10 and promotes ferroptosis in ovarian cancer cells [104, 105]. SCD1 and fatty acid binding protein 4 (FABP4) contribute to the resistance to ferroptosis during tumor recurrence [106]. Moreover, it has been shown that SCD1 is also involved in the regulation of ferroptosis in several cancers, including gastric, ovarian, and colon cancers [107–109]. Members of the SREBP family that are involved in cholesterol metabolism also participate in the regulation on ferroptosis [110]. In some cancers, activation of the PI3K/AKT/mTOR signaling pathway resists ferroptosis through controlling the activities of sterol regulatory element-binding proteins 1 (SREBP1) and SCD1. Activation of PI3K/AKT/mTOR signaling pathway affects the binding between SREBP1 and mTOR, further activating downstream SCD1 to promote lipid synthesis to defend against ferroptosis [108]. SREBP2 reduces both intracellular iron content and lipid peroxidation through transcriptionally regulating transferrin (TF), thereby resisting ferroptosis [111]. Ferroptosis can be promoted when the peroxisome proliferators-activated receptors (PPARs) activity is affected by the p53 regulator MDM2 and MDMX [112–115]. PPARα also regulates ferroptosis by affecting FABP1 [116]. Besides, lipid molecules impacts ferroptosis [117, 118]. Energy metabolism genes such as AMPK play a vital role in the regulation of tumor ferroptosis [119, 120]. Therefore, tumor ferroptosis can be effectively controlled through metabolism regulation (Fig. 3).

Ferroptosis is inseparable from lipid peroxidation. Glutathione as an important reductant in organisms plays a critical role in the process of anti-oxidation. Several substances that are either related to glutathione are known to be involved in ferroptosis [121, 122]. Consumption of glutathione is one of the necessary conditions for ferroptosis [123, 124]. Glutamine is one of the raw materials for the synthesis of glutathione. Attenuated glutamine catabolism inhibits ferroptosis and reduces its damaging effect on cardiomyocytes [125]. Glutathione is converted intracellularly to GSSG to resist ferroptosis. In addition to regulating glutathione levels, the cellular uptake of cysteine can also be targeted to promote or inhibit ferroptosis. Cysteine deprivation in the mouse pancreatic ductal adenocarcinoma model leads to ferroptosis and inhibits tumor cell growth [121]. The incidence of ferroptosis is increased significantly after the exchanges between cystine and glutamate inhibited by the anticancer drugs [126]. Many cysteine transporters, such as Cysteinyl-tRNA synthetase (CARS) and SLC7A11, involve in tumor ferroptosis [122, 127]. Meanwhile, the stability and expression of SLC7A11 is affected by OUTB1 and BAP1 to promote or inhibit tumor ferroptosis [128, 129]. Therefore, Glutathione is an important target to regulate tumor growth associated with ferroptosis [130–132]. Taken together, we may be able to extend the effects of lipid metabolism and glutamine metabolism on ferroptosis to other metabolic types through these intersections to identify additional ferroptosis regulatory targets.

### The function of metabolism and pyroptosis during tumorigenesis

**Fig. 4:**
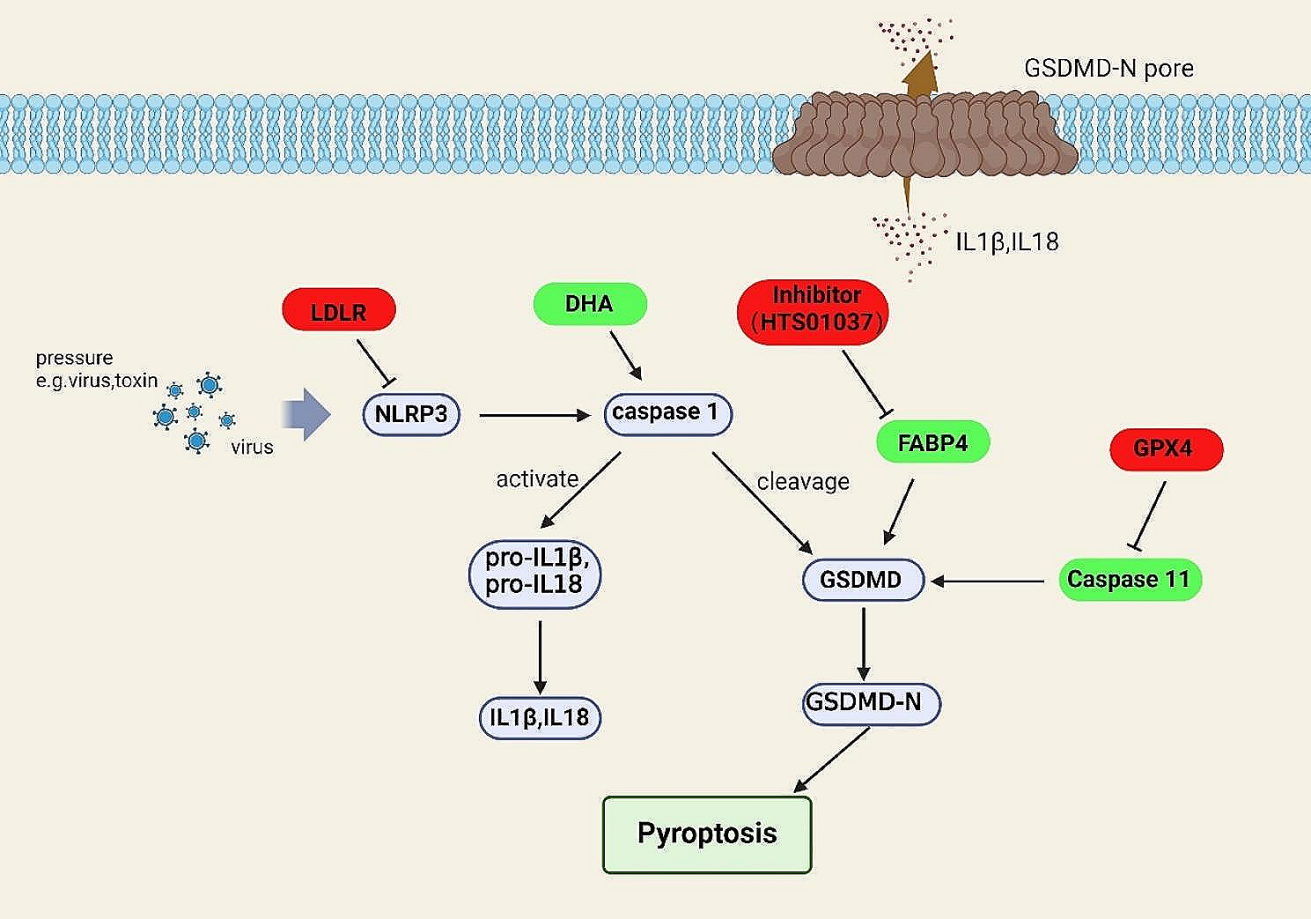
Metabolites, metabolic pathways and related metabolic genes that play the roles in pyroptosis. NLRP3 plays an important role in the process of pyroptosis, which inhibition of NLRP3 will lead to the downregulation of pyroptosis. LDLR inhibits pyroptosis by mediating NLRP3. DHA promotes pyroptosis through affecting caspase1, and FABP4 exert the same effect through activating GSDMD. GPX4 inhibit pyroptosis by affecting the processing of GSDMD by caspase11, respectively. The red boxes represent negative regulators and the green boxes represent positive regulators

Pyroptosis is defined as inflammatory cell death, which depends on the Gasdermin family of proteins and inflammatory caspase. Many factors can induce pyroptosis, such as bacterial, viral infections, and energy stress et al. [133]. Lipids such as docosahexaenoic acid (DHA) change the GSDMD activity to influence pyroptosis [134]. During pyroptosis, some proteins related to lipid metabolism, such as Fatty acid binding protein 4 (FABP4), play a role [135]. GPX4 is related to lipid peroxidation and also regulates pyroptosis. Loss of GPX4 upregulates the generation of GSDMD N-terminals by activating caspase11, then promotes pyroptosis [136]. The low-density lipoprotein receptor (LDLR) and lipids related to cholesterol transport play a role in the regulation of tumor pyroptosis [137]. LDLR negatively regulates the activity of the inflammasome NLRP3 and inhibit the inflammatory response, while the absence of LDLR is conducive to pyroptosis (Fig. 4) [138].

Energy stress is one of the most significant cellular environment factors that induces the cells to enter into pyroptosis [139, 140]. For example, the protein SGLT2, related to glucose transport, is involved in the regulation of NLRP3 activity [140]. In addition to glucose, fatty acids also take part in the regulation of inflammasome activity [141]. NLRP3 is regulated by metabolism-related substances, but is also involved in obesity-related inflammation [142]. Pyroptosis occurs not only in tumors but also in infectious and metabolic diseases. As a regulated death, current interest in pyroptosis is mainly in inflammatory vesicles and members of the gasdermin family. The role of metabolic regulation in pyroptosis has been gradually uncovered, and many metabolites also participate in inflammatory response. It is expected more findings will be demonstrated that metabolism influences tumorigenesis by regulating pyroptosis.

### The cuproptosis is a new role of metabolism during tumorigenesis

**Fig. 5:**
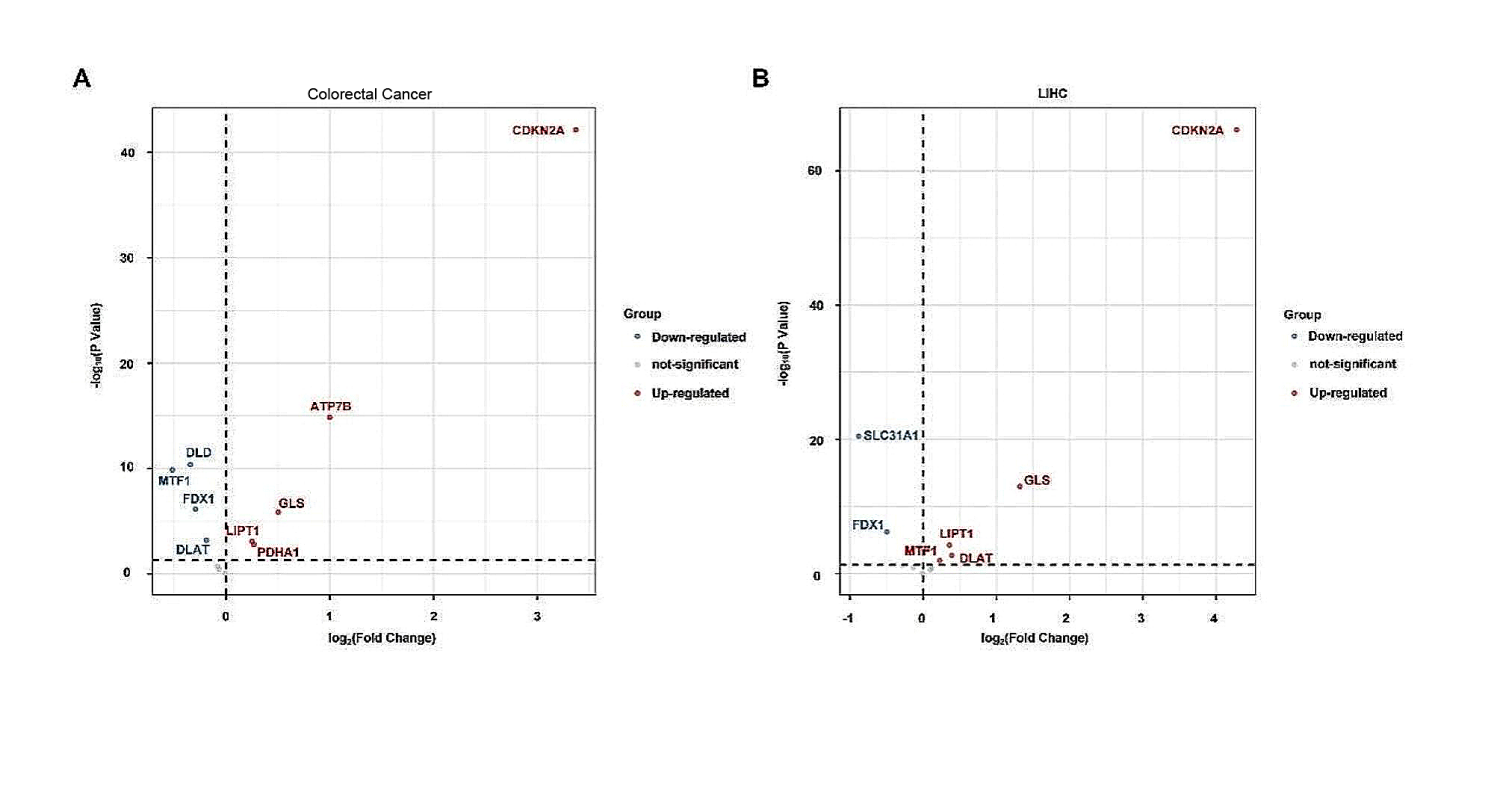
The expression levels of cuproptosis genes in colorectal cancer and hepatocellular carcinoma. (A and B) The expression levels of cuproptosis genes in colorectal cancer (A) and hepatocellular carcinoma (B). The blue dot represents the genes that are down-regulated in cancer, the red dot means these genes are up-regulated in cancer (*p* < 0.05), and those with no significant difference compared with normal tissues are indicated in gray. Both mRNA Seq data and clinical data collected from TCGA database, including COAD, READ and LIHC reveal that the COAD and READ are merged into colorectal cancer based on gene names. In the mRNA differential expression analysis, the R package Deseq2 was used to differential expression analysis. The genes with a fold change (FC) > 0 and an adjusted P-value (FDR) > 0.05 were retained for further analysis

**Fig. 6:**
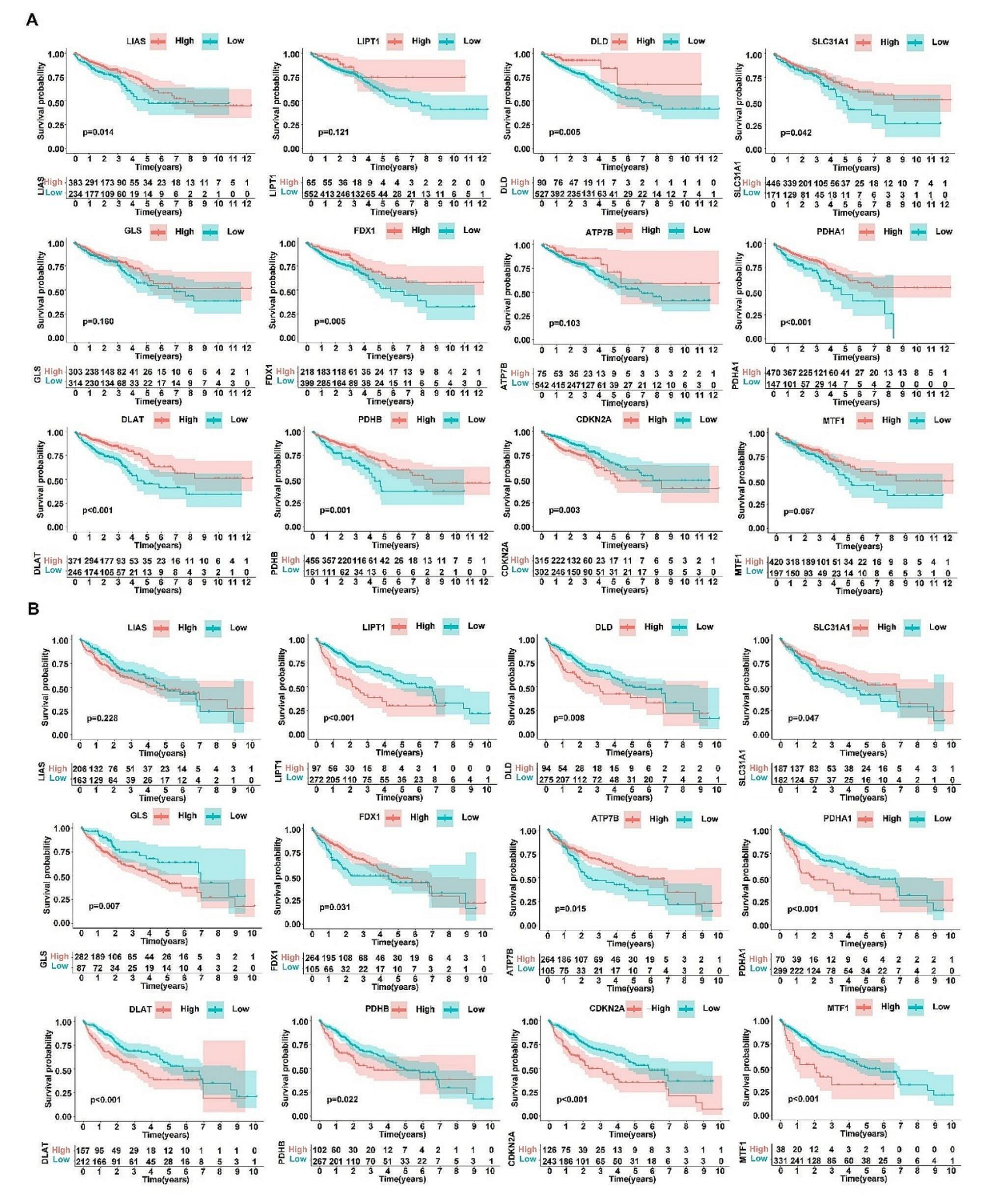
Survival analyses of cuproptosis-related genes in both colorectal cancer and hepatocellular carcinoma. (A and B) The survival analysis of cuproptosis-related genes in colorectal cancer (A) and hepatocellular carcinoma (B). The top part of the survival analysis for each gene shows the Kaplan-Meier survival curves for the genes obtained by the optimal division method, with the red and blue lines representing the high and low expression groups based on gene expression levels, and the horizontal coordinate (Time(years)) representing the survival time and the vertical coordinate (Survival probability) representing the survival rate. In the bottom part of the graph, the horizontal coordinate Time (years) represents the follow-up time, and the optimal division method divides all patients into high and low expression groups at the beginning of the follow-up period. The mRNA expression data of genes and corresponding clinical survival data across colorectal cancer and LIHC were merged for expression survival analysis. Tumor samples were divided into high and low groups according to median gene expression value. The R package survival was used to fit the survival time and survival status for the two groups. Differences in P value were examined in the survival outcomes of the groups according to Kaplan–Meier survival analysis

Copper death has been recently defined as a new type of cell death [143]. It is induced under the action of the copper ionophore elesclomol [143, 144]. During the process, excessive accumulation of copper ions promotes the oligomerization of fatty acylated proteins dihydrolipoamide Sacetyltransferase (DLAT) and FDX1-related Fe-S cluster proteins in the tricarboxylic acid cycle [143]. The Fe-S cluster proteins associate with FDX1 to become the important components of the electron transport chain [145]. Cuproptosis is down-regulated after the function of ETC suppressed by respiratory chain complexes I and III inhibitors [146], suggesting that ETC is another copper death-regulating target in addition to TCA [143, 147]. After cuproptosis is formally defined, several types of relationships between cuproptosis and tumorigenesis have been discovered. For example, the lncRNAs analysis that are related to cuproptosis to obtain the relevant expression profiles is an effective way to determine the possibility of prognosis [148, 149]. Recent studies include thatcuproptosis-related genes are also associated with cancer prognosis [150–152]. In particular, we performed the differential expression analysis and survival analysis of cuproptosis-related genes in colorectal cancer and hepatocellular carcinoma, and found that there are different expression and survival outcomes (Figs. 5 and 6). Since cuproptosis is dependent on the accumulation of copper ions and lipid-acylated proteins in the tricarboxylic acid cycle, we might be able to regulate the tricarboxylic acid cycle by modulating intracellular copper ion metabolism [143, 153]. Therefore, controlling the occurrence and development of a particular metabolic process can regulate the production of specific intermediates and result in an effect on the tricarboxylic acid cycle and cuproptosis.

### Autophagy derived from metabolic activities during tumorigenesis

**Fig. 7:**
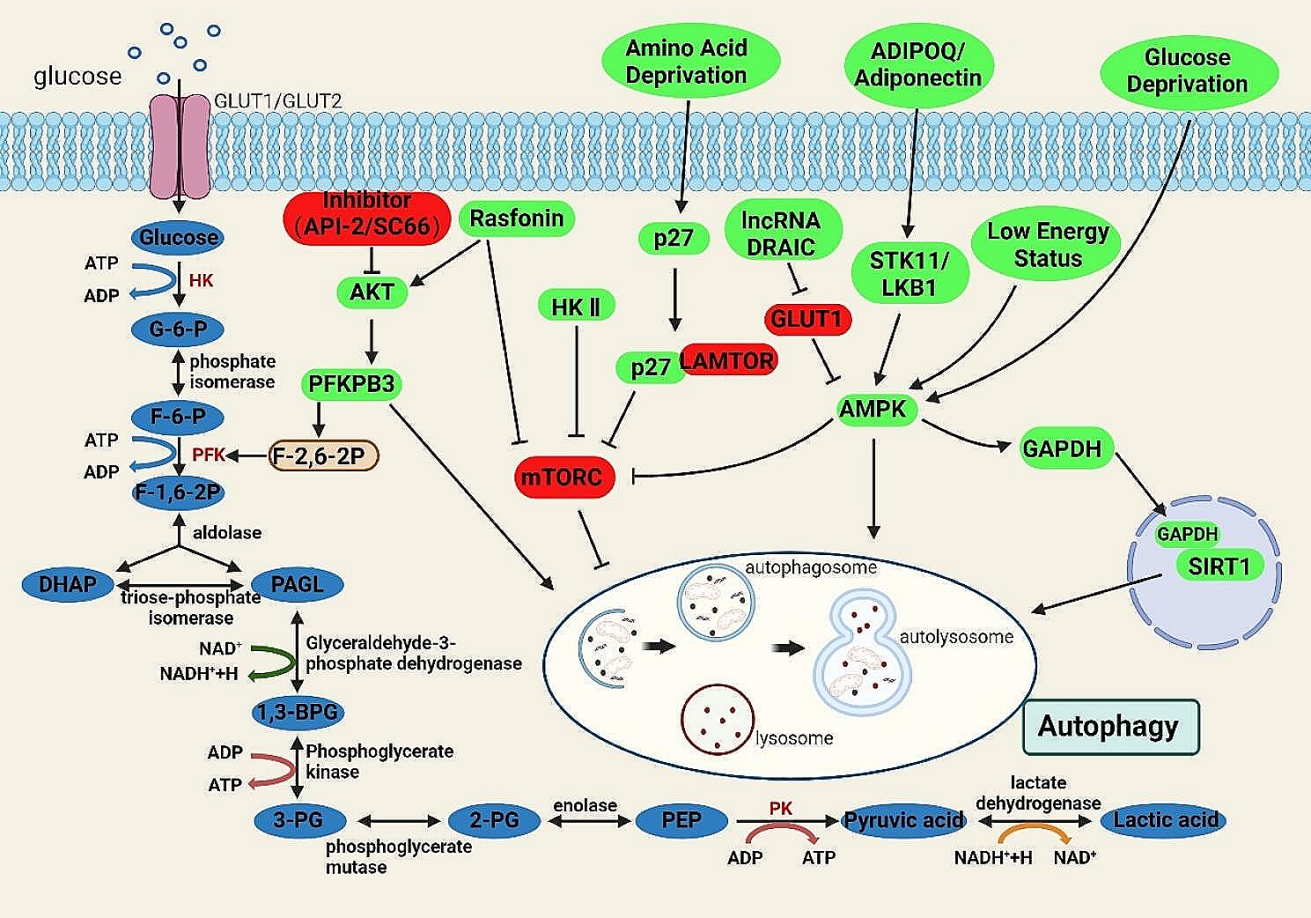
Metabolites, metabolic pathways and related metabolic genes that work in autophagy. mTORC is an important negative regulator of autophagy, and many factors promote autophagy by inhibiting mTORC. Rasfonin regulates autophagy both through AKT and mTORC. Amino acid deprivation inhibits mTORC through p27, its combination with LAMTOR in turn accelerates autophagy. Hexokinase II can inhibit mTORC, thus promoting autophagy. AMPK can also inhibit mTORC under the regulation of lncRNA DRAIC. Under low energy state stimulation of AMPK can promote autophagy. The STK11/LKB1-AMPK axis also exerts a pro-autophagic effect by affecting AMPK. Glucose deprivation promotes nuclear translocation of GAPDH and binding to SITR1 via AMPK, which in turn promotes autophagy. The red boxes represent negative regulators and the green boxes represent positive regulators

Autophagy is defined as a process that is lysosome dependent, which is featured by the generation of autophagosome [9]. Autophagosomes then fuse with lysosomes to form autolysosomes, and lysosome-carried enzymes can finally hydrolyze the material inside the autophagosome [154]. Since autophagy is also a process of energy reuse, it can be upregulated in response to the needs of cellular life activities under certain conditions, such as starvation and glucose deprivation [155]. During the process related to glucose metabolism, Akt is associated with various enzymes in glycolysis to influence autophagy. Inhibition of PFKFB3 expression in the presence of Akt inhibition attenuates the rasforin-induced autophagy in gastric cancer cells [156]. Besides, Akt also affects autophagy through regulating the activity of hexokinase [157]. Hexokinase-II inhibits mTOR to regulate glucose deprivation induced autophagy [158]. mTOR is an important negative regulator of autophagy, and it is also involved in the regulation of various metabolic activities, especially insulin-mediated glucose metabolism [159]. Promotion or inhibition autophagy of amino acids by regulating mTOR is a proven truth [160, 161]. Amino acids depletion inhibits mTOR through p27, thereby promotes autophagy [162]. AMPK also takes part in tumor autophagy [163]. AMPK phosphorylates mammalian autophagy-related Unc-51-like kinase (ULK1) under low-energy conditions to promote autophagy [164, 165]. In addition, large numbers of non-tumor cells are present in the tumor microenvironment. These cells can secrete various factors such as growth factors and immune factors. One of the adipocyte products, ADIPOQ/adiponectin, activates AMPK via STK11/LKB1. The activated AMPK can further promote autophagy in breast cancer cells by activating ULK1 [166]. In the absence of glucose, AMPK also mediates GAPDH nuclear transfer to activate Sirt1 and promotes autophagy (Fig. 7) [167].

The function of amino acids in autophagy has been thoroughly reported in recent years. Loss of glutamine promotes two important activities to lead to autophagy, including the acetylation of PGK1, and the phosphorylation of Beclin1 [168]. During cancer radiation therapy, cancer cells that are resistant to glutamine depletion can induce autophagy through activating ATG5 to defend against radiation-induced damage [169]. In addition to glutamine, other amino acids can also regulate autophagy [170–175], suggesting that tumor autophagy and tumorigenesis can be effectively regulated by changing amino acid levels or metabolic processes in vivo.

Besides the glucose and amino acids, the lipid affected autophagy regulation also has been found recently [176, 177]. The AMPK/mTORC signaling pathway plays an important role in the lipid-related autophagy, indicating that the lipid-regulated tumor autophagy could also associate with AMPK/mTORC [178–181]. Besides, autophagy can reversely affect lipid metabolism under various situations. For example, autophagy causes degradation, formation, and accumulation of lipid droplets [182]. Deletion of autophagy-related genes induces intracellular lipid accumulation in non-small cell lung cancer models [183]. In addition, autophagy-related genes such as ATG5 and ATG7 are also involved in metabolic regulation and metabolism-related tumor therapy [184–187]. Taken together, autophagy and autophagy-associated proteins are regulated in a variety of metabolic situations and can likewise control metabolism in tumorigenesis.

### Metabolism, cell death and tumorigenesis

As one of the most important physiological activities of cells, metabolism not only regulates cellular energy status, structure and function, but also participates in a variety of important intracellular modifications. Generally, metabolic stress and metabolite-related transporters can involve in tumor regulation. Energy supplies the basis power for all life activities, but tumors have higher energy requirements than normal tissues [188]. Glucose transporters effectively alter the processes of tumor cell glucose metabolism and regulate tumorigenesis [33, 189, 190]. The amino acid transporters are also associated with tumorigenesis. Cystine transporter SLC7A11 transports cystine across the cell membrane to participate in glutathione biosynthesis to regulate tumorigenesis [191, 192].

Metabolism is also closely linked to post-translational modifications such as glycosylation, palmitoylation, and lactylation. Different types of glycosylations activate or stabilize the function of biomolecules and regulate tumorigenesis and tumor growth [193–195]. Previous studies have discovered that palmitoylation affects the development of melanoma, lung cancer and other tumors [196–198]. Lactylation can likewise affect tumorigenesis and progression [199–201]. Hypersuccinylation, another product of glucose metabolism, is associated with the TCA cycle to inhibit mitochondrial respiration and induces apoptosis and tumor development [202].

Metabolism and cell death are the two vital life activities in cells. For example, cuproptosis, a newly defined cell death model, contributes to cell death by affecting the TCA cycle [203]. Copper ion is an essential trace element, which is not only related to intracellular oxidation-reduction property, but also acts as one of the main triggers of cuproptosis. Copper ions implicates a variety of intracellular activities, such as redox reactions and the mitochondrial respiratory chain. Disturbances in copper ion metabolism leads to the well-known Wilson’s disease [204], on the other hand, depletion of copper ions in mitochondria is associated with tumor suppression [205]. Ferroptosis is another trace element-dependent cell death closely related to amino acid metabolism [206]. Ferroptosis also requires phospholipid peroxide that is a product of lipid metabolism and redox reaction [206], and through excessive accumulation of lipid peroxide it causes cell death.

The secondarily considered link between metabolism and cell death is that multiple cell death regulators are also the energy sensors. AMPK is an important intracellular energy receptor. AMPK activates downstream molecules and promotes energy production after it either senses a rise in the ADP/ATP ratio [207], or inhibits energy-consuming processes such as the synthesis of biomolecule glycogen. AMPK implicates in metabolic diseases as an important energy sensor and regulator [208]. Energy stress is a potent inducer of cell death, and RIPK1 induces cell death under energy stress, whereas AMPK inhibits RIPK1 and metabolic stress-induced RIPK1-associated cell death by phosphorylation [209]. mTOR is also a very important autophagy regulatory molecular [210–212]. AMPK/mTOR together regulate tumorigenesis and tumor progression in both lung and colorectal cancers [213, 214], demonstrating their combined contribution to metabolism and cell death for tumorigenesis.

Metabolites are the intersection between metabolism and cell death, which also modulate cell death. For example, α-KG, an intermediate of the TCA cycle, recruits pro-caspase-8 and GSDMC in acidic environments and activates pyroptosis [137]. Certain metabolites released by cell death also perform functions to modulate peripheral immune or inflammatory responses [215, 216]. Metabolites released by apoptosis can alter the gene expression of myeloid cells and attenuate inflammatory responses [217]. Some cell death releases inflammatory substances into the environment and causes severe inflammatory response. NLRP3 inflammasome activation, such as IL-1β and IL-18 production, promotes immune cell infiltration in the tumor microenvironment and enhances chemotherapy-induced anti-tumor immunity [218, 219].

Regulated Cell Death (RCD) used to be considered non-immunogenic. However, an Immunogenic Cell Death (ICD) related to tumor immunity has been proposed after sufficient investigations with more research on cell death patterns [220]. ICD occurs with Damage-Associated Molecular Patterns (DAMPs) release or exposure of some immunogenic substances during cell death [220, 221]. A series of stresses such as radiation, viruses and ischemia promote DAMPs release into the extracellular environment [221]. As an effective immune activator, DAMPs then combine with its receptor and further promote inflammation [220]. ICD is thought to modulate the tumor immune microenvironment and is one of the targets for tumor immunotherapy [222]. Many cell death models exert immunogenic potential in tumors and influence tumorigenesis and development by activating the tumor immune response.

Immune system can activate ferroptosis. Interferon gamma (IFNγ) released by CD8^+^T cells in the tumor microenvironment restrains cystine uptake, and tumor cells deficient in cystine have difficulty in resisting lipid peroxidation and thus promote ferroptosis [223]. In turn, ferroptosis that occurs in tumor cells acts inversely on the immune system and promotes the development of immune cells [224]. Cell death models such as ferroptosis, pyroptosis and cuproptosis can influence the immune infiltration of TME [225]. These cell death patterns affect immune cell infiltration [219] and expression levels of immune checkpoint molecules [226], ultimately regulating tumor survival and progression by activating or suppressing the tumor immune response.

### TME, metabolism and cell death

One of the most significant effects of TME on tumor metabolism is that it regulates the rate of tumor cell glucose metabolism. In oxygen-deficient TME, the PI3K/Akt/HIF-1α signaling axis is activated and further regulates the process of glycolysis [227], and the alteration of HIF-1α and glycolysis promote the proliferation of tumor cells. The high rate of glycolysis in tumor cells leads to the production of large amounts of lactate, which is transported outside the cell and then accumulates in the environment leading to a decrease in pH and creating an acidic tumor microenvironment [228]. A low pH environments has been demonstrated that can induce necrosis and apoptosis [229]. Since there are many different types of cells present in the TME, the buildup of lactate can regulate the function of these cells. Previous studies demonstrates that lactate promotes M2 macrophage polarization in TME and facilitates pituitary adenoma (PA) invasion [230]. Tumor-associated macrophages are a class of cells with high presence in the tumor microenvironment and are thought to be associated with the regulation of cellular metabolism [231, 232]. It is thought to be associated with the regulation of iron levels [233], while high iron content is considered to be another conducive condition for ferroptosis. Other immune cells enriched in the TME and their immune functions are also regulated by lactate, which leads to tumor immune escape by altering the immune environment. Firstly, high levels of lactate are inherently immunosuppressive [200]. The acidic environment caused by lactate interferes with the lactate metabolism of T cells via monocarboxylate transporter 1 (MCT1) and inhibits the proliferation of T cells [234]. Moreover, the absence of MCT1 in Treg cells not only inhibits tumor growth, but also accompanies the high expression of immune checkpoint molecules [235]. Secondly, lactate represses T cell by modulating the expression of immune checkpoint molecules PD-1 and PD-L1, which causes immune escape. Lactate restrains PD-1 expression on effector T cells to suppress T cell cytotoxicity, which causes immune escape and even affects the effectiveness of tumor immunotherapy [236–238]. Lactate in the tumor microenvironment also acts as an upstream molecule for the transcription factor NF-κB, and synergistically affect tumor angiogenesis [239].

Lipids are closely related to tumor immunity and TME. Lipids accumulated in the TME can regulate anti-tumor immunity [240, 241]. In TME, hypoglycemia and hypoxia promote fatty acid catabolism in CD8^+^T cells and enhance its anti-tumor capacity [242]. Besides, obesity can also change the TME and the immune cell. High-fat diet changes lipids composition in TME, alters CD8^+^T cell fatty acids uptake and reduces the function and number of CD8^+^T cell. Finally harm the anti-tumor immunity [243]. Lipids also link TME and cell death together. For example, cholesterol in the TME upregulates CD36 expression in CD8^+^T cell, leads to CD8^+^T cell ferroptosis and inhibits its antitumor efficacy [244]. Tryptophan and CD8 + T cells also synergistically promote tumor cell apoptosis, thereby inhibiting tumor cell growth [245].

Transcription factors are involved in the regulation of a wide range of physiological activities, and there are many transcription factors with known functions that regulates metabolism, e.g., PPARs can participate in the regulation of lipid metabolism [246] and c-Myc can modulate glutamine metabolism [247]. c-Myc upregulates glutaminase (GLS) expression to promote glutamine catabolism [248], while glutamine deficiency induces myc-dependent apoptosis [249]. Transcription factors have an important role in the regulation of cell death. The first manifestation is in the promotion or inhibition of the cell death process. For example, the transcription factor Dlx2 suppresses canonical TGFβ signaling in tumor cells thereby inhibiting apoptosis [250]. Secondly, transcription factors shifts the type of cell death such as ATF3 converts hepatocyte apoptosis to necrotic apoptosis by regulating RIPK3 expression [251].

### Targeting metabolism to induce cell death for tumor therapy

Compared to traditional chemotherapy and radiotherapy, cancer targeting therapy is more precise and has less side effects on the normal tissue [252–254]. Metabolism affects tumor development and plays a role in tumor therapy and prognosis. For example, the blockade of purine metabolism affects tumor cell proliferation and induces tumor death through inhibiting nucleic acid synthesis [255]. Or, we could also regulate tumor death by targeting intracellular carbon metabolism [256]. Meanwhile, current therapeutic approaches targeting cell death has made a good progress, proving that cell death can also be an effective tumor therapy target. Targeting cell death related-molecules such as Bcl-2 family proteins and NLRP3 effectively represses tumor growth [257, 258]. In addition, NLRP3, as an important inflammasome, is involved in immune checkpoint-related tumor immunotherapy [259]. We have summarized the drugs and molecules that can influence cell death in tumors (Tables 1, 2, 3, 4, 5 and 6). In addition to targeting metabolism and cell death respectively, many studies have now elucidated that targeting the interplay between them is a good strategy for cancer therapy [109, 260]. Taken together, with the in-depth study of the relationship between different metabolisms and cell death, we can treat tumors more effectively in the future.

## Conclusion

The wide spectrum of research on tumor metabolism has been developing for decades, including glucose metabolism, lipid metabolism, nucleotide metabolism, etc. For the cell death occurrence, the metabolic change-induced energy stress is one important reason. With the research in progress, knowledge on metabolic stress as an important influencing factor in tumors has been expanded. A variety of metabolism-related enzymes are related to tumor development and can be referenced for clinical therapy. Cell death is strongly linked to energy metabolism [261–263]. In particular, moreattentionhas been paidto the relationship between cell death and metabolism in tumors, as metabolism is found to be an effective way to mediate cell death [264–266]. Both energy production efficacy and capacities to produce the intermediates as signaling molecules for intracellular activities can be changed through following aspects, including affecting the enzymatic activities, regulating the levels of various key products from metabolic processes and the nutrients intake into cells [267, 268]. On the other hand, changes in metabolism also playsan important role in the induction of cell death in a variety of tumors [34, 37, 167]. These studies elucidate how cell death is regulated and affected by various metabolic pathways, and their roles during tumorigenesis and progression. Nevertheless, there are still many undiscovered regulatory relationships between metabolism and cell death that await for future studies.

Each of these cell death modes are regulated by cancer metabolism and can be involved in the regulation of tumorigenesis. Various metabolic processes promote or inhibit cell death not only through directly exerting stimuli that cause stress and cell death but also by affecting various important regulators of different cell death regulatory processes. In addition to these, the tumor microenvironment is also involved in the regulation of cell death by metabolism. Under certain circumstances, due to changes in intracellular metabolic profile and cell death, the tumor microenvironment will be affected accordingly. It indicates that it is feasible and effective to regulate tumorigenesis by modulating the tumor metabolism-cell death network. In the future studies, we may identify more targets associated with tumor metabolism to affect tumor cell death and discover more ways to regulate tumorigenesis and tumor therapy.

**Table 1:** Selected drugs or molecules associated with apoptosis in oncology research, their effects on apoptosis and targets of action

Cell death	Medicine	Inhibition or promotion	Target	References
Apoptosis	Smac/Diablo	Promotion	IAP	[269, 270]
Apoptosis	Sulforaphane	Promotion	Bax/Bak	[271]
Apoptosis	Cisatracurium	Promotion	lncRNA-p21	[272]
Apoptosis	Obatoclax	Promotion	Bcl-2, Survivin, Wnt/β-catenin pathway	[273, 274]
Apoptosis	NO.0449 − 0145	Promotion	APE1	[275]
Apoptosis	Curcumin	Promotion	Caspase8/9/3	[276, 277]
Apoptosis	Diosmetin	Promotion	STAT3/c-Myc pathway	[278]
Apoptosis	z-VDVAD-fmk, z-IETD-fmk	Inhibition	Caspase2/8	[279]
Apoptosis	Tauroursodeoxycholic acid	Inhibition	ER stress	[280]

**Table 2:** Selected drugs or molecules associated with necrosis in oncology research, the effects on necrosis and targets of action

Cell death	Medicine	Inhibition or promotion	Target	References	
Necrosis	Tetrathiomolybdate	Promotion	Inhibit angiogenesis	[281]	
Necrosis	Simvastatin, Metformin	Promotion	Ripk1, Ripk3	[282]	
Necrosis	Melatonin	Promotion	Bcl2/Bax	[283]	
Necrosis	CuZnSOD, MnSOD	Inhibition	Inhibit ROS generate	[284]	

**Table 3:** Selected drugs or molecules associated with necroptosis in oncology research, their effects on necroptosis and targets of action

Cell death	Medicine	Inhibition or promotion	Target	References	
Necroptosis	NO.0449 − 0145	Promotion	APE1	[275]	
Necroptosis	CBL0137	Promotion	ZBP1	[86]	
Necroptosis	BV6	Promotion	TNF-α	[285]	
Necroptosis	Shikonin	Promotion	PKM2	[286]	
Necroptosis	Necrostatin-1	Inhibition	RIP1	[287]	
Necroptosis	NBC1	Inhibition	Hsp70	[288]	

**Table 4:** Selected drugs or molecules associated with ferroptosis in oncology research, their effects on ferroptosis and targets of action

Cell death	Medicine	Inhibition or promotion	Target	References	
Ferroptosis	Erastin	Promotion	System Xc^-^	[98]	
Ferroptosis	Brequinar	Promotion	DHODH	[289]	
Ferroptosis	Nortriptyline hydrochloride	Promotion	RBMS1	[290]	
Ferroptosis	Erianin	Promotion	Ca2+/CaM	[291]	
Ferroptosis	RSL3	Promotion	GPX4, NF-κB	[292, 293]	
Ferroptosis	Tagitinin C	Promotion	PERK-Nrf2-HO-1	[294]	
Ferroptosis	Metformin	Promotion	SLC7A11	[295]	
Ferroptosis	Dihydroartemisinin	Promotion	AMPK/mTOR/p70S6k	[296]	
Ferroptosis	Elesclomol, copper	Promotion	ATP7A	[297]	
Ferroptosis	Cetuximab	Promotion	NRF2/HO-1	[298]	
Ferroptosis	EF24	Promotion	HMOX1	[299]	
Ferroptosis	Flubendazole	Promotion	P53	[300]	
Ferroptosis	Simvastatin	Promotion	HMGCR	[301]	
Ferroptosis	Apatinib	Promotion	SREBP-1α, GSH	[302]	
Ferroptosis	Ferrostatin-1, Liproxstatin‑1	Inhibition	lipid peroxidation	[98, 303]	
Ferroptosis	Dihydroartemisinin	Inhibition	PERK/ATF4/HSPA5	[304]	

**Table 5:** Selected drugs or molecules associated with pyroptosis in oncology research, their effects on pyroptosis and targets of action

Cell death	Medicine	Inhibition or promotion	Target	References	
Pyroptosis	NO.0449 − 0145	Promotion	APE1A	[275]	
Pyroptosis	Metformin	Promotion	AMPK/SIRT1/NF-κB	[305]	
Pyroptosis	Paclitaxel, Cisplatin, Miltirone, Tetraarsenic hexoxide	Promotion	GSDME	[306–308]	
Pyroptosis	α-KG	Promotion	DR6	[137]	
Pyroptosis	Val-boroPro	Promotion	CARD8	[309]	
Pyroptosis	Benzimidazoles	Promotion	NF-κB/NLRP3/GSDMD	[310]	
Pyroptosis	Dihydroartemisinin	Promotion	AIM2/Caspase-3/ DFNA5	[311]	
Pyroptosis	Metformin	Promotion	FOXO3	[312]	
Pyroptosis	Disulfiram, Cucurbitacin B	Inhibition	GSDMD	[313, 314]	

**Table 6:** Selected drugs or molecules associated with autophagy in oncology research, their effects on autophagy and targets of action

Cell death	Medicine	Inhibition or promotion	Target	References	
Autophagy	Carfilzomib, ONX 0912	Promotion	ATF4	[315]	
Autophagy	Dihydromyricetin	Promotion	ROS/STAT3	[316]	
Autophagy	Rapamycin, Latcripin-7 A, Metformin, RAD001	Promotion	mTOR	[317–320]	
Autophagy	Narciclasine	Promotion	AMPK/ULK1	[321]	
Autophagy	PX-866	Inhibition	PI3K	[322]	

### Electronic supplementary material

Below is the link to the electronic supplementary material.

Supplementary Material 1

Supplementary Material 2

## Data Availability

All data supporting Figs. 5 and 6 are available within the supplementary tables.

## Abbreviations

PD-1: programmed cell death protein 1
PD-L1: programmed cell death ligand 1
CTLA-4: cytotoxic T-lymphocyte antigen-4
GLUT4: glucose transporter 4
ISL1: Insulin gene enhancer protein 1
MC1R: melanocortin-1 receptor
EGFR: epidermal growth factor receptor
TCA cycle: tricarboxylic acid cycle
LKB1: liver kinase B1
RIPK1: Receptor-interacting protein kinase 1
mTOR: mammalian target of rapamycin
α-KG: α-ketoglutarate
GSDMC: gasdermin C
RCD: regulated cell death (RCD)
ICD: immunogenic cell death
DAMPs: damage-associated molecular patterns
IFNγ: interferon gamma
TME: tumor microenvironment
PI3K: phosphoinositide 3-kinase
HIF-1α: Hypoxia-inducible factor-1alpha
MCT1: monocarboxylate transporter 1
PPARs: peroxisome proliferator-activated receptors
GLS: glutaminase
LAT1: L-type amino acid transporter 1
OX-LDL: oxidized low-density lipoprotein
ACAT1: cholesterol acyltransferase 1
FASN: fatty acid synthase
NAMPT: icotinamide phosphoribosyltransferase
GLUT1: glucose transporter 1
ACLY: ATP citrate lyase
ACC1: Acetyl CoA carboxylase 1
α-KG: α-ketoglutarate
AKT: protein kinase B
F-1,6-2P: fructose-1,6-bisphosphate
TIGAR: TP53-induced glycolysis and apoptosis regulator
ROS: reactive oxygen species
ASCT2: alanine-serine-cysteine transporter 2
GLN: glutamine
BAX: bcl2 associated X protein
BAK: bcl2 antagonist/killer 1
TRADD: TNF receptor-associated death domain
PERK: protein kinase RNA-like endoplasmic reticulum kinase
mtDNA: mitochondrial DNA
ZBP1: Z-form nucleic acid binding protein 1
GLTP: glycolipid transfer protein
DMF: dimethyl fumarate
MLKL: mixed lineage kinase domain-like protein
DHA: docosahexaenoic acid
RIPK1: Receptor-interacting protein kinase 1
RIPK3: Receptor-interacting protein kinase 3
CARS: cysteinyl-tRNA synthetase
SREBP1: sterol-regulatory element binding protein 1
SREBP2: sterol-regulatory element binding protein 2
TF: transferrin
FABP1: fatty acid binding protein 1
PPARα: peroxisome proliferators-activated receptorα
MDM2: murine double minute2
MDMX: murine double minute
SCD1: stearoyl-CoA desaturase-1
CoQ10: coenzyme Q10
AMPK: adenosine 5‘-monophosphate (AMP)-activated protein kinase
SLC7A11: solute carrier family 7 member 11
GPX4: glutathione peroxidase 4
LDLR: low-density lipoprotein receptor
FABP4: fatty acid binding protein 4
NLRP3: NOD-like receptor thermal protein domain associated protein 3
IL-1β: interleukin-1β
IL-18: interleukin-18
GSDMD: gasdermin D
DHA: docosahexaenoic acid
G-6-P: glucose-6-phosphate
F-6-P: fructose-6-phosphate
F-1,6-2P: fructose-1,6-bisphosphate
DHAP: dihydroxyacetone phosphate
PGAL: 3-phosphoglyceraldehyde
3-PG: 3-phosphoglycerate
2-PG: 2-phosphoglycerate
PEP: Phosphoenolpyruvate
HK: hexokinase
PFK: phosphofructokinase
GAPDH: glyceraldehyde-3-phosphate dehydrogenase
SIRT1: sirtuin 1
mTORC: mammalian target of rapamycin complex
TAK1: TGFβ-activated kinase 1
ULK1: Unc-51-like kinase 1
TF: transferrin
